# The AMSlide for noninvasive time‐lapse imaging of arbuscular mycorrhizal symbiosis

**DOI:** 10.1111/jmi.13313

**Published:** 2024-05-15

**Authors:** Jennifer McGaley, Ben Schneider, Uta Paszkowski

**Affiliations:** ^1^ Department of Plant Sciences Crop Science Centre, University of Cambridge Cambridge UK; ^2^ Department of Biochemistry and Biophysics Stockholm University Stockholm Sweden

**Keywords:** arbuscular mycorrhizal symbiosis, fungi, live, microscopy, time‐lapse

## Abstract

Arbuscular mycorrhizal (AM) symbiosis, the nutritional partnership between AM fungi and most plant species, is globally ubiquitous and of great ecological and agricultural importance. Studying the processes of AM symbiosis is confounded by its highly spatiotemporally dynamic nature. While microscopy methods exist to probe the spatial side of this plant–fungal interaction, the temporal side remains more challenging, as reliable deep‐tissue time‐lapse imaging requires both symbiotic partners to remain undisturbed over prolonged time periods. Here, we introduce the AMSlide: a noninvasive, high‐resolution, live‐imaging system optimised for AM symbiosis research. We demonstrate the AMSlide's applications in confocal microscopy of mycorrhizal roots, from whole colonisation zones to subcellular structures, over timeframes from minutes to weeks. The AMSlide's versatility for different microscope set‐ups, imaging techniques, and plant and fungal species is also outlined. It is hoped that the AMSlide will be applied in future research to fill in the temporal blanks in our understanding of AM symbiosis, as well as broader root and rhizosphere processes.

## LAY DESCRIPTION

In most soils across the globe, you will find arbuscular mycorrhizal (AM) fungi. AM fungi act like an extended root system, foraging in the soil for nutrients and delivering them back to their plant‐partner, while the plant feeds them sugars and fats. This partnership is important for soil health, nutrient cycling and plant nutrition. But it is not an easy interaction to study: AM fungi are microscopic, growing in the soil but also deep inside plant roots, all concealed underground. Additionally, the interaction is very dynamic, with fungal structures and partners constantly appearing, growing and disappearing. To overcome these challenges, we developed imaging chambers, named AMSlides, that allow mycorrhizal structures and processes to be revealed by high‐resolution microscopy without disturbing plant or fungus. Consequently, the AM partnership can be reliably observed over time. This work highlights some example findings from the AMSlides and discusses future applications in AM research and beyond.

## 1 INTRODUCTION

Arbuscular mycorrhizal (AM) symbiosis is a nutritional mutualism between a group of filamentous soil fungi (Glomeromycotina) and plants.^1^ AM symbiosis occurs on all continents, in all soils, and in nearly all plant species, with roles in nutrient cycling, carbon storage, soil stability and plant health and nutrition.^2^, ^3^, ^4^, ^5^, ^6^, ^7^ Aligned with this global abundance and importance, researchers strive to better understand the molecular, cellular, physiological and ecological aspects of AM functioning.^8^, ^9^, ^10^

During AM symbiosis, the fungal partner extends a network of hyphae into the soil while simultaneously colonising plant roots, growing between and within plant root cells. Inside the root cortical cells, AM fungal hyphae form specialised nutrient exchange structures: tree‐like ‘arbuscules’ and/or knot‐like ‘coils’.^11^ Here, the AM fungi receive lipids and sugars from their plant host, necessary to their survival as obligate biotrophs, and reciprocate with water and mineral nutrients gathered from beyond the reach of the plant's own roots.^12^ Later in the symbiosis, fungal storage organs (vesicles) and reproductive structures (spores) develop.

Spatially, this partnership is highly heterogeneous: there can be a mosaic of symbiotic structures and developmental stages in a single colonised zone of a root, multiple colonised zones and fungal individuals per root, and networks made up of different root and hypha types.^11^, ^13^, ^14^, ^15^, ^16^ Additionally, there is temporal dynamism: the arbuscules develop and collapse within just a few days, there is constant progression or contraction of hyphal colonisation throughout the roots as well as out into the soil, there can be turnover in fungal symbionts, and all this takes place in the fluid backdrop of a growing root system.^16^, ^17^, ^18^, ^19^, ^20^

Microscopy has been a fundamental tool in AM research. Since compound light microscopy aided the discovery of AM fungi at the turn of the 19th century, a multitude of microscopy techniques have been employed to reveal the spatial aspects of AM symbiosis.^21^, ^22^, ^23^, ^24^ To highlight a few applications, brightfield microscopy of stained roots is routinely used to quantify the extent of AM fungal colonisation in plant roots, while fluorescence microscopy coupled with dyes has revealed the morphologies and chemical aspects of symbiotic structures.^25^, ^26^, ^27^, ^28^, ^29^, ^30^ Increasingly, fluorescence‐based microscopy of genetically encoded reporters is used to characterise where AM‐relevant genes are expressed and proteins are localised during the symbiosis.^18^, ^31^, ^32^, ^33^, ^34^ Further, the development of fluorescence‐based biosensors allows distributions of molecules and processes to be mapped.^35^, ^36^, ^37^, ^38^ More recently applied microscopy techniques include X‐ray microscopy for in situ elucidation of fungal networks inside and outside roots,^39^ and spatial transcriptomics, mapping sequenced transcripts onto brightfield micrographs of roots hosting AM fungi.^40^

However, the temporal side of AM symbiosis remains challenging. To accurately follow AM processes, plant and fungal partners must remain alive and undisturbed over lengthy timeframes, such as the multiple days of arbuscule lifespan, and weeks of fungal life cycle.^17^, ^18^ But this symbiosis manifests within and around roots, all concealed in opaque soil, poorly compatible with live, light‐based microscopy. Time‐lapses have been performed on excised roots, but they remain limited in duration and reliability due to the substantial root damage.^38^, ^41^ Longer imaging has been achieved via intact roots grown in ‘membrane sandwiches’ or pots, but the disturbance caused by uprooting and transferring them to microscope slides curtails the time‐lapse timeframe to minutes to hours.^35^ In situ imaging negates the need to disrupt plant or fungus, but the bulk of such studies make use of artificial biological systems, such as root organ cultures (in which the plant‐partner lacks a shoot), or do not include a plant host at all.^16^, ^42^, ^43^, ^44^, ^45^, ^46^, ^47^, ^48^ It remains to be seen how representative these systems are of a natural underground environment.

To our knowledge, the pioneering works of Kobae et al.^18^, ^27^, ^49^ represent the only examples of long‐term, noninvasive imaging of AM symbiosis within soil‐grown plant roots. Using a glass‐bottomed Petri dish, rice plants expressing arbuscule‐localised fluorescent reporters were cocultivated with AM fungi. Imaging through the glass ‘window’ captured the appearance and collapse of arbuscules and dynamics of lipid droplets.^18^, ^27^, ^49^ Widefield epifluorescence was predominantly used, capturing long‐term colonisation dynamics but with limited subcellular resolution. The few instances employing confocal laser scanning microscopy ran for shorter periods, up to 19 h, with resolution restricted by the available microscope hardware of the time (over a decade ago).^27^, ^49^ Consequently, at the resolution now possible and desirable in AM microscopy, there remains a temporal *terra incognita*.

This technical note presents the AMSlide: a noninvasive, high‐resolution, live‐imaging system optimised for studying AM symbiosis. We demonstrate how AMSlides can be used to:

- reliably monitor mycorrhizal symbiosis in rice roots without impacting plant growth or AM colonisation,
- obtain a high‐resolution view of roots colonised by AM fungi, from large scale colonisation zones to subcellular domains of the arbuscules,
- follow AM symbiosis over the timeframes of seconds, hours, and days to weeks, unveiling arbuscule development and collapse dynamics, arbuscule lifespan, and the rate of colonisation progression.

We also show how, on the technical side, AMSlides:

- are cheap, adaptable, and functional with most confocal set‐ups,
- open the door to time‐intrinsic imaging techniques such as fluorescence recovery after photobleaching (FRAP),
- are compatible for studies involving other plant and AM fungal species,
- can be used in non‐AM applications.

It is hoped the AMSlides will be adopted to bring the important temporal context to AM research, including AM signalling, arbuscule development, and nutrient exchange. They could facilitate characterisation of symbiotic dynamics with different plant–fungal partnerships or environmental conditions, enabling a more representative view into complex AM symbioses. Further, AMSlides could allow broader root‐ and rhizosphere‐related processes, including root development, immunity and soil community interactions, to be temporally resolved.

## 2 MATERIALS AND METHODS

### 2.1 Plant and fungal material

Mycorrhizal rice experiments were performed with a genetically encoded fluorescent reporter line from Kobae et al.,^49^ in which the AM‐specific gene *OsSCAMP* is tagged with eGFP under its native promoter (‘*eGFP‐SCAMP*’ from here onwards). eGFP‐SCAMP localises to the plasma membrane and peri‐arbuscular membrane of colonised root cells, thereby allowing intracellular hyphae and arbuscules to be visualised.^49^

Nonmycorrhizal rice AMSlide experiments were performed with a genetically encoded fluorescent reporter line from Bureau et al.,^50^ which coexpresses the plasma membrane marker *eCFP‐Lti6a* under the *ZmUBI1* promoter and histone marker *H2B‐mCherry* under the *CsCMV* promoter (‘*eCFP‐Lti6a;H2B‐mCherry*’ from here onwards).

*Nicotiana benthamiana* mycorrhizal experiments utilised the *GFP16C* reporter line from Ruix et al.,^51^ whereby *mGFP5* is constitutively expressed under the *CaMV35S* promoter (*GFP16C* from here onwards).

Spores of the AM fungus *Rhizophagus irregularis* (DAOM197198) were extracted from an axenic coculture with carrot root organ cultures (*Daucus carota* L.), as described by Bécard and Fortin.^52^ *Gigaspora margarita* (BEG34) was applied as crude inoculum from pot‐culture with *Tagetes patula*, established and maintained as described by Habte and Osorio.^53^

### 2.2 Preparation of AMSlide chambers

AMSlide chambers were designed in the online CAD platform Onshape^54^ and 3D printed using a Cura S3 (Ultimaker, Utrecht, the Netherlands) (3D print Files S1–S5). Black PETG filament was used for the chamber structure, with natural PVA to generate water‐soluble supports (Ultimaker, Utrecht, the Netherlands). 24 × 50 mm #1.5H coverslips (THORLABS, Newton, New Jersey, USA) were sealed to the chambers using transparent 732 silicon sealant (DOW, Midland, Michigan, USA). AMSlides were washed with reverse‐osmosis (RO) water before use. Detailed information on assembly is available in the Supplementary Protocol (File S6).

### 2.3 Germination and inoculation of rice seedlings

De‐husked rice seeds were surface sterilised using 3% hypochlorite solution for 15 min, before rinsing with sterile water and incubating on 0.6% bactoagar plates in the dark at 30°C for 5 days.

Rice seedlings could be precolonised using a nurse‐plant system, or directly inoculated with AM fungi upon planting in the AMSlide chamber. Nurse plants were created by inoculating rice seedlings with 200 *R. irregularis* spores in black ⌀60 mm Petri dishes in silica sand. Nurse plants were grown for 4 weeks until highly colonised. Pregerminated rice seedlings were planted alongside the established nurse plants and cocultivated for 12 days. Direct inoculation with *R. irregularis* involved mixing 100 spores into the sand for each AMSlide. Direct inoculation with *G. margarita* consisted of 5% (v/v) crude inoculum in sand.

### 2.4 Planting and growth in AMSlides

**FIGURE 1:**
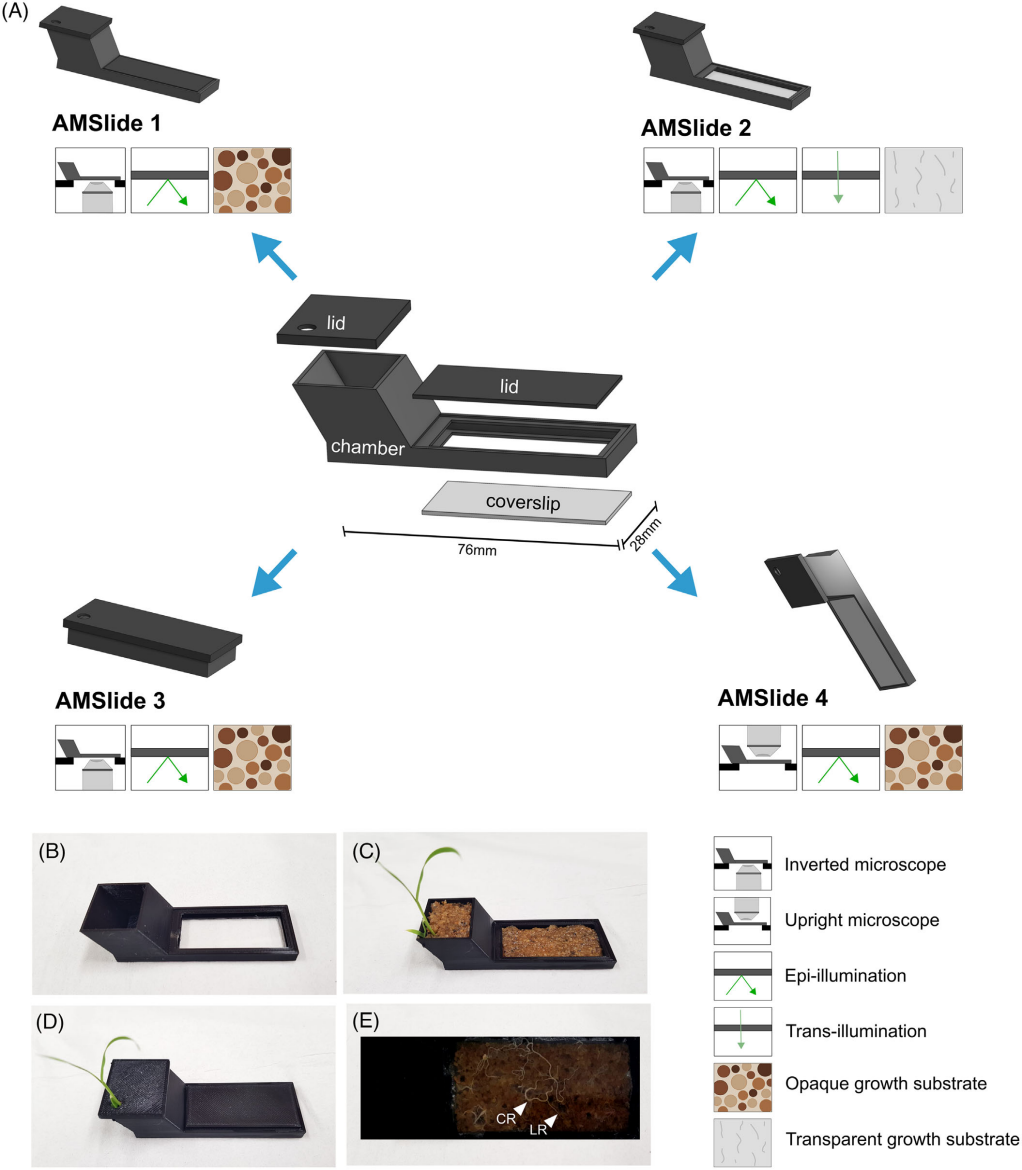
Overview of AMSlide chamber designs and planting set‐up. (A) Schematic of different AMSlide chamber versions with their microscopy features (symbol key on bottom right of figure). AMSlides 1, 2 and 3 all have coverslips on the lower side of the chamber, lids on the upper, and are grown at 30° angle. AMSlide 4 has a coverslip on the upper side, lid sealed to the lower side, and is grown vertical. 3D renders obtained from Onshape.^54^ (B–D) Steps of planting rice into AMSlide 1: (B) empty chamber with coverslip attached, (C) sand and rice seedling added, and (D) covered with lids. (E) Example root growth in AMSlide 1 at 7 days postplanting, with crown root (CR) and lateral root (LR) annotated.

Rice seedlings were planted into AMSlide chambers containing sand with or without inoculum (depending on precolonisation). *N. benthamiana* seeds were directly applied to the surface of inoculated sand. Lids were added according to AMSlide type, as detailed in the Supplementary Protocol (File S6) and Figure 1. AMSlides 1–3 were grown on a 30° slope to guide downwards root growth. AMSlide 4 was grown in a vertical orientation to guide root growth along the ‘upper’ coverslip (Figure 1A).

For the comparative growth‐check experiment, *eGFP‐SCAMP* rice seedlings were planted in sand in 120 mm tall cones and ⌀60 mm black Petri dishes containing 200 spores and 300 spores, respectively (spore number scaled up to respective pot volume).

Rice plants were watered with RO water until sand was saturated three times per week for the first week. Subsequently, RO water and half‐strength Hoagland's solution (modified low *P*_i_ = 25 µM) were alternated. *N. benthamiana* seeds were kept damp with RO water for the first 3 weeks, then received alternating half‐strength low *P*_i_ Hoagland's and RO water as above. Both plant species were grown under 12 h days of 300 uE light, 28°C, relative humidity 65%, and nights of 20°C.

### 2.5 Imaging AMSlides

A Stellaris 8 Falcon confocal microscope (Leica Microsystems GmbH, Wetzlar, Germany) was used for all imaging. A 10× dry objective and LED fluorescence lamp were used to locate regions of interest before switching to a 40× water immersion objective for imaging. A white light laser was used at 4% power to excite GFP at 488 nm, with detection by hybrid detectors at 500–550 nm (GFP) and 600–700 nm (endogenous autofluorescence). eCFP was excited at 440 nm, with detection at 470–520 nm, and mCherry was excited at 585 nm, with detection at 600–650 nm. Where applicable, transmitted brightfield light was detected with a PMT detector.

During imaging, stage *x*/*y* coordinates were recorded to facilitate refinding and imaging of regions of interest after desired time periods. LasX Navigator (Leica Microsystems GmbH, Wetzlar, Germany) was used to obtain merged tile‐scans of entire roots. Between imaging, AMSlides were returned to the growth chamber.

### 2.6 Image analysis

All image processing and analysis was performed in FIJI/ImageJ^55^ (details available in Supplementary Protocol File S6).

### 2.7 Harvesting, staining and quantifying mycorrhizal colonisation

To assess growth and colonisation in AMSlides, rice plants were harvested at 20 and 30 days after inoculation (with *R. irregularis*). Roots and shoots were washed, surface‐dried and their fresh weights were recorded. Roots were stained with Trypan Blue and colonisation quantified as previously described.^32^ Brightfield images of stained roots were acquired with a GXM‐L2800 microscope (GT Vision, Wickhambrook, UK).

## 3 RESULTS

### 3.1 Growth and AM colonisation of rice plants in AMSlides is comparable to conventional pot systems

To ensure that data from rice grown and imaged in AMSlides was representative, a comparative experiment was conducted between AMSlide 1, AMSlide 3 and two conventional rice‐growth set‐ups: 120 mm cones^56^ and ⌀60 mm black Petri dishes.^32^ *eGFP‐SCAMP* rice plants grown in each chamber type were harvested at the early timepoints of 20 days after inoculation and 30 days after inoculation to capture the establishment of AM symbiosis. To test any effects of confocal laser scanning microscopy, half of the AMSlide chambers were imaged daily between the timepoints, and half were not.

Rice plants grown in the AMSlides showed comparable growth and level of AM colonisation to conventional pot systems. Shoot and root development appeared normal in the AMSlides (Figure S1), and there was no significant difference in shoot or root fresh weight, colonisation level or AM morphology between plants grown in AMSlide 1, AMSlide 3, cone or Petri systems at either timepoint (Figure S2). Similarly, no significant difference in growth or AM colonisation was observed between AMSlide‐grown plants that experienced daily time‐lapse imaging and those that did not (Figures S1 and S2).

### 3.2 Colonised rice roots can be noninvasively imaged at high resolution

Colonised rice roots were observed in AMSlides from 7 days postplanting for precolonised seedlings, 12 days postplanting for crude‐inoculated seedlings, and 20 days postplanting for spore‐inoculated seedlings. Roots tracked along the bottom coverslips, meaning the focal distance required was equal to excised, slide‐mounted roots and well within the working distance of the objectives. As such, fungal structures 3–4 cell layers deep could be imaged (Figure S3).

**FIGURE 2:**
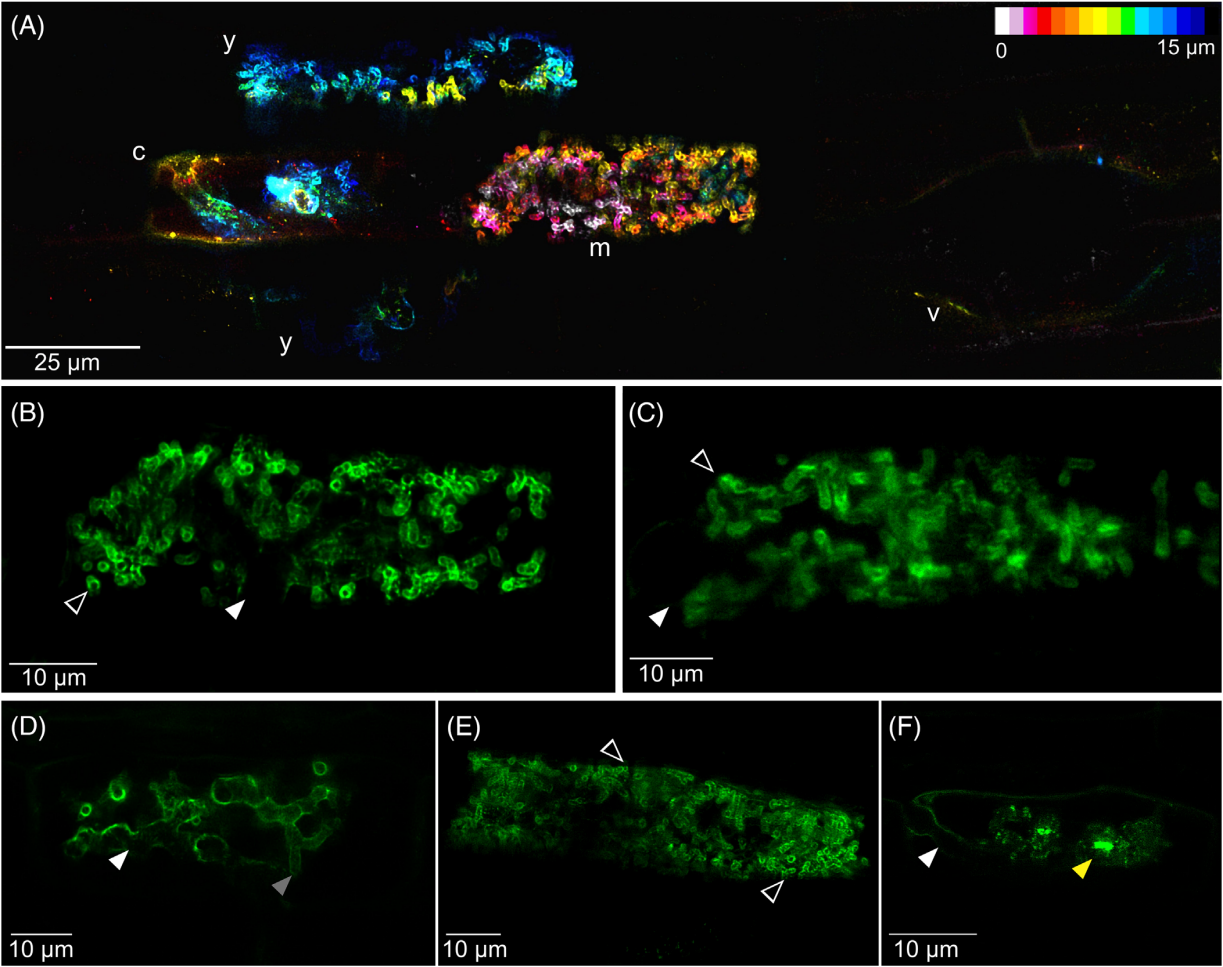
High‐resolution confocal laser scanning microscopy of symbiotic structures in *R. irregularis* inoculated *eGFP‐SCAMP* rice grown in AMSlides. (A) Example colonisation zone in AMSlide 3, including young arbuscules, y, mature arbuscule, m, collapsed arbuscule, c, and a vesicle, v. Maximum intensity projection of 15 µm, coloured by depth. (B) High magnification of a young arbuscule using AMSlide 3. (C) Comparative high magnification of a young arbuscule in a pot‐grown, excised root. (D–F) Example micrographs of (D) young, (E) mature, and (F) collapse developmental stages of arbuscules imaged in AMSlide 3 with subcellular features annotated. Green = eGFP, white solid arrowhead = arbuscule trunk, grey arrowhead = arbuscule coarse branch, open arrowhead = arbuscule fine branch, yellow arrowhead = eGFP‐SCAMP puncta.

AM structures outlined by eGFP‐SCAMP were noninvasively imaged at high resolution (Figure 2A–C). Arbuscules at each developmental stage could be clearly observed: young arbuscules consisting of a trunk and coarse branches, not filling the cell (Figure 2D), mature arbuscules with fine branches, filling the cell (Figure 2E), and collapsed arbuscules, with clumped plant–fungal material and the indicative SCAMP punctae^49^ (Figure 2F). AM colonisation was imaged from the scale of an entire colonisation zone (Figure S4), to the individual colonisation structures (Figure 2A), down to the subcellular domains (Figure 2B–F).

### 3.3 AM dynamics can be captured over many time scales

**FIGURE 3:**
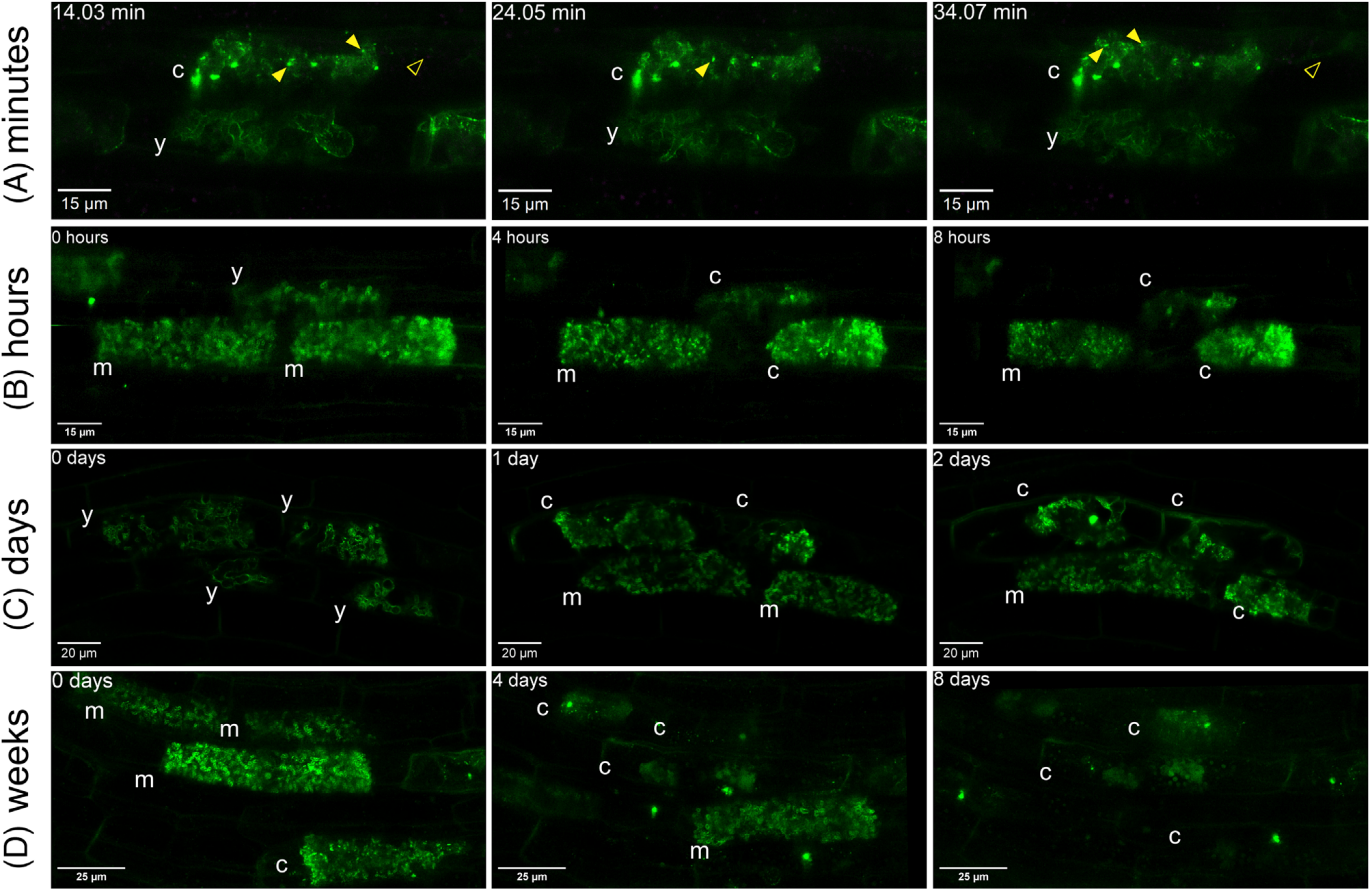
Dynamics of AM symbiosis monitored over a broad range of time scales. Time‐lapse snapshots of symbiotic structures in *R. irregularis‐*colonised *eGFP‐SCAMP* rice plants in AMSlide 3, imaged at (A) 1‐min intervals for 45 min, (B) 4‐h intervals for 12 h, (C) 24‐h intervals for 3 days and (D) daily intervals for over a week. All micrographs are maximum intensity projections of the relevant arbuscule volume. Green = eGFP. Yellow solid arrowheads = mobile eGFP‐SCAMP puncta, yellow open arrowheads = transvacuolar strands, y = young arbuscule, m = mature arbuscule, c = collapsing/collapsed arbuscule.

Due to the noninvasive nature of the AMSlide, the temporal dynamics of AM symbiosis could be followed over a range of relevant time scales, from minutes to over a week. High‐frequency scanning over minutes captured subcellular dynamics in cells hosting arbuscules of different developmental stages, for example, a young and a collapsing arbuscule in Figure 3A (Movie S1). This revealed highly mobile transvacuolar strands and SCAMP punctae around collapsing arbuscules, and smaller mobile bodies in cells hosting young arbuscules.

The AMSlides also revealed the longer‐term processes of arbuscule development and collapse over hours. For example, Figure 3B and Movie S2 show a collapsing arbuscule imaged every 4 h for 12 h. Arbuscule branches withdrew, SCAMP punctae appeared and plant–fungal material formed clumps.

When imaged daily, the AMSlides enabled slower processes to be monitored. The confocal‐level resolution allowed arbuscule expansion and branching (Figure 3C, Movie 1), and vesicle development (Movie S3) to be captured. The longest time‐lapses performed lasted 9 days, showing asynchronous arbuscule development and collapse, and cell recolonisation (Figure 3D, Movie 2). Arbuscule lifetime could be measured, and while variable, initial development generally took 1–2 days, followed by a 1–2 day (but sometimes as long as 4 day) period in a cell‐filling state. Arbuscule collapse was rapid, mostly taking place within a day, but requiring a further 3‐ to 5‐day period to completely disappear from the cell.

**MOVIE 1:** Timelapse movie capturing development and collapse of arbuscules. Arbusculated cells in *R. irregularis*‐colonised *eGFP‐SCAMP* rice roots in AMSlide 3 were imaged at daily intervals for 3 days. Micrographs are maximum intensity projections. Green = eGFP.

**MOVIE 2:** Timelapse movie capturing colonisation dynamics over a prolonged time period. Region of *eGFP‐SCAMP* rice root colonised by *R. irregularis*, grown in AMSlide 3, was imaged at daily intervals for 9 days. Micrographs are maximum intensity projections. Green = eGFP.

By mapping the arbuscules as they appeared at a colonisation front (the growing edge of a colonised zone), the rate of colonisation progression through roots could be measured (Figure S4a). In the example shown in Figure S4, arbuscule appearance progressed along the root at a steady rate of around 400 µm per day.

### 3.4 AMSlides open the door to time‐intrinsic imaging techniques

The ability to monitor colonised roots over prolonged time periods unlocks further imaging techniques, such as fluorescence recovery after photobleaching (FRAP). FRAP can be used to detect protein movement as well as distinguishing actively translated protein pools from residual, stable pools. The latter technique requires longer imaging than is compatible with excised roots.

**FIGURE 4:**
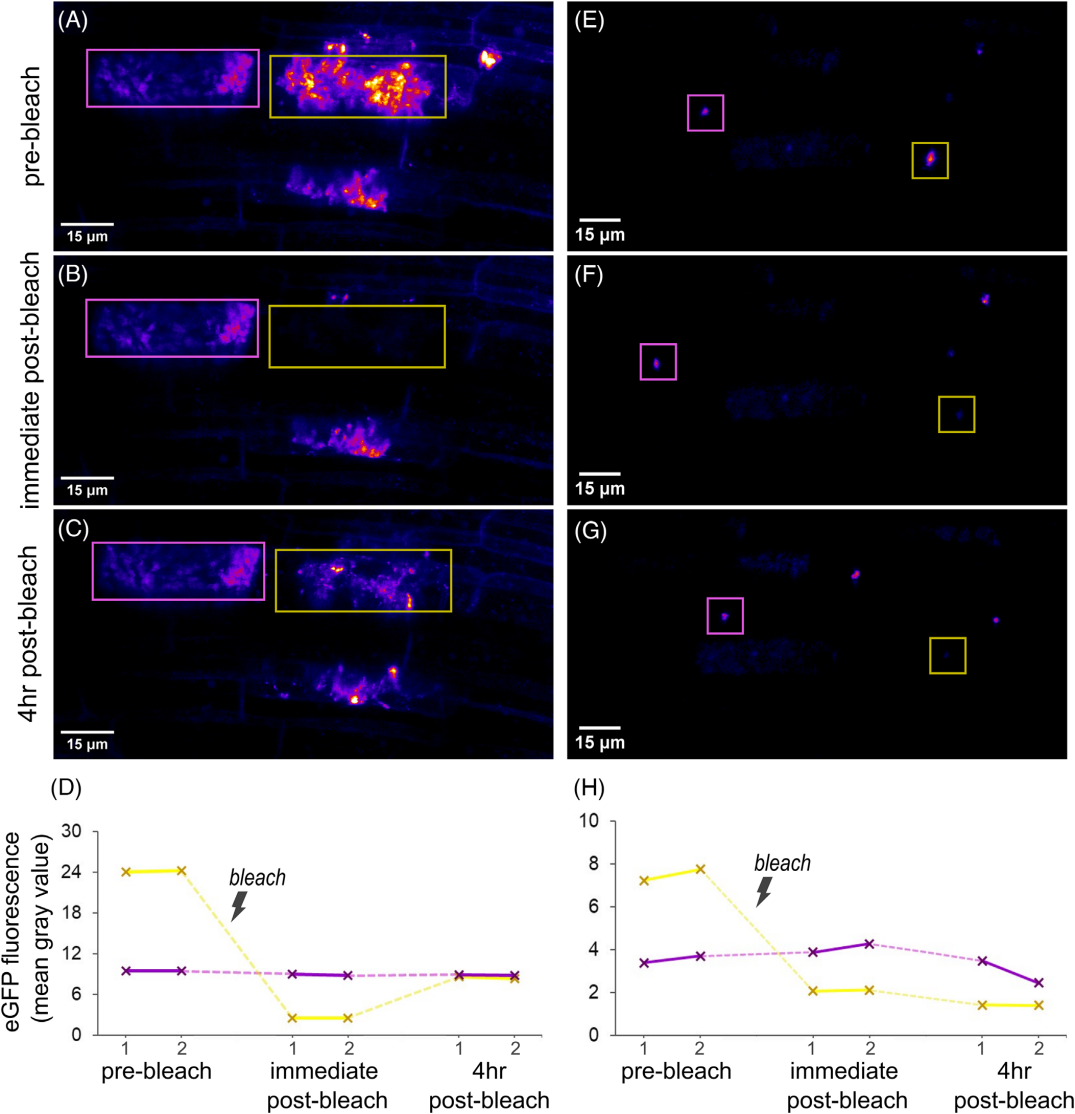
AMSlides can facilitate long‐term FRAP experiments. *eGFP‐SCAMP*‐expressing rice plants were colonised by *R. irregularis* in AMSlide 3 chambers. Regions hosting (A–C) young arbuscules or (E–G) collapsed arbuscules were imaged before bleaching, immediately after bleaching the eGFP of one arbuscule (yellow square), and 4 h after bleaching, with two *z*‐stacks per timepoint. Normal imaging was performed with 4% laser power, bleaching was performed with 100% laser power. The mean eGFP intensity of ROIs in maximum intensity projections of (D) young arbuscules and (H) collapsed arbuscules was measured to quantify fluorescence recovery. Yellow = bleached ROI, magenta = control ROI.

Using the AMSlide, long‐term FRAP was achieved. In the example in Figure 4, the eGFP of eGFP‐SCAMP was bleached in cells hosting young arbuscules (Figure 4A–C) and cells hosting collapsed arbuscules (Figure 4E–G). After 4 h, fluorescence recovery was observed around the young arbuscules, implying active production of SCAMP at this arbuscule stage, while no recovery was seen in cells hosting collapsed arbuscules, suggesting SCAMP punctae represent a stable pool of SCAMP that is not being replenished (Figure 4D and H).

### 3.5 AMSlides are compatible with different equipment, plant species and AM fungi

**FIGURE 5:**
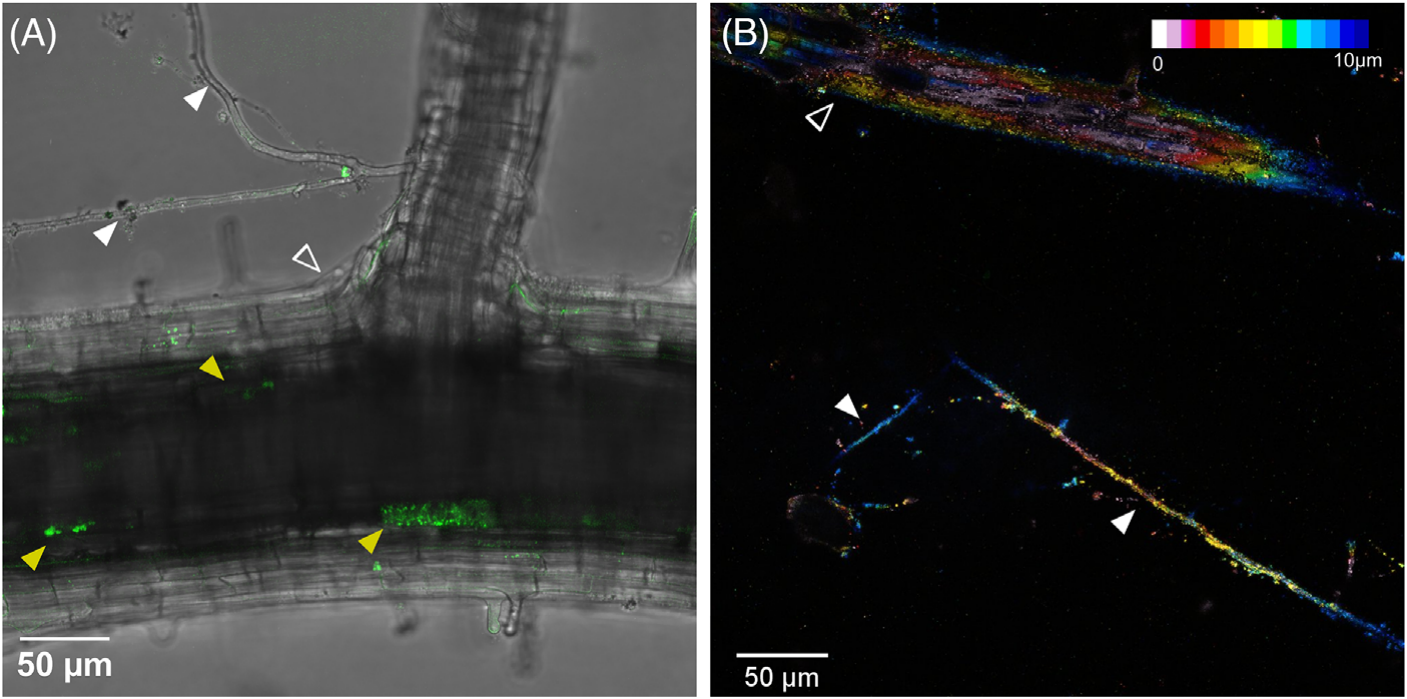
External hyphae of AM fungi imaged using AMSlides. The external hyphae of *R. irregularis* in coculture with *eGFP‐SCAMP* rice plants in AMSlide 1 could be visualised using (A) transmitted brightfield light after washing sand from above coverslip (green = eGFP, grey = brightfield, maximum intensity projection of 10 µm volume), or (B) by epifluorescence excitation of endogenous hyphal autofluorescence with 527 nm excitation and 532–616 nm detection (coloured by depth, maximum intensity projection of 10 µm volume). White solid arrowheads = AM fungal hyphae, open arrowheads = rice root, yellow solid arrowheads = eGFP‐SCAMP signal around arbuscules.

While imaging of mycorrhizal rice roots in sand was mostly carried out using AMSlides 1 and 3 on an inverted confocal microscope, AMSlide 4 is compatible with upright confocal microscopy, and AMSlide 2 could enable transmitted light microscopy for plants that engage with mycorrhiza in transparent media^57^ (File S6). By gently washing away the sand on top of the coverslip of AMSlide 1, brightfield and epifluorescence coimaging enabled simultaneous visualisation of AM fungi within rice roots (e.g. arbuscules in Figure 5A) and outside the roots (e.g. hyphal networks emanating from roots, Figure 5A). External hyphae in sand‐filled AMSlides 1 and 3 could also be imaged by epi‐illumination thanks to their endogenous autofluorescence (Figure 5B).

AMSlides are also compatible with different partner species. Additional to the interaction between rice and *R. irregularis*, time‐lapse imaging captured the dynamics of rice colonised by *Gigaspora margarita*, and *Nicotiana benthamiana* colonised by *R. irregularis* (Figure S5). It is already known that *G. margarita* follows a different colonisation strategy to *R. irregularis*, forming many intracellular coils in addition to arbuscules.^58^ The AMSlide revealed further temporal differences, such as far higher longevity of coils (persisted throughout the duration of all time‐lapses) compared to arbuscules, but a loss of *eGFP‐SCAMP* from around coils after 1–2 days (Movie 3, Figure S5). Arbuscule lifespan appeared similar between rice and *N. benthamiana* colonised by *R. irregularis* (Figure S5); however, an AM‐specific fluorescent reporter in *N. benthamiana* would improve this quantification.

**MOVIE 3:** Timelapse movie of *G. margarita* colonised *eGFP‐SCAMP* rice root. Cells hosting coils (and later, arbuscules) in AMSlide 1 were imaged at daily intervals for 3 days. Micrographs are maximum intensity projections of entire coil/arbuscule volume. Green = eGFP, magenta = autofluorescence.

### 3.6 AMSlides can be used in nonmycorrhiza applications

**FIGURE 6:**
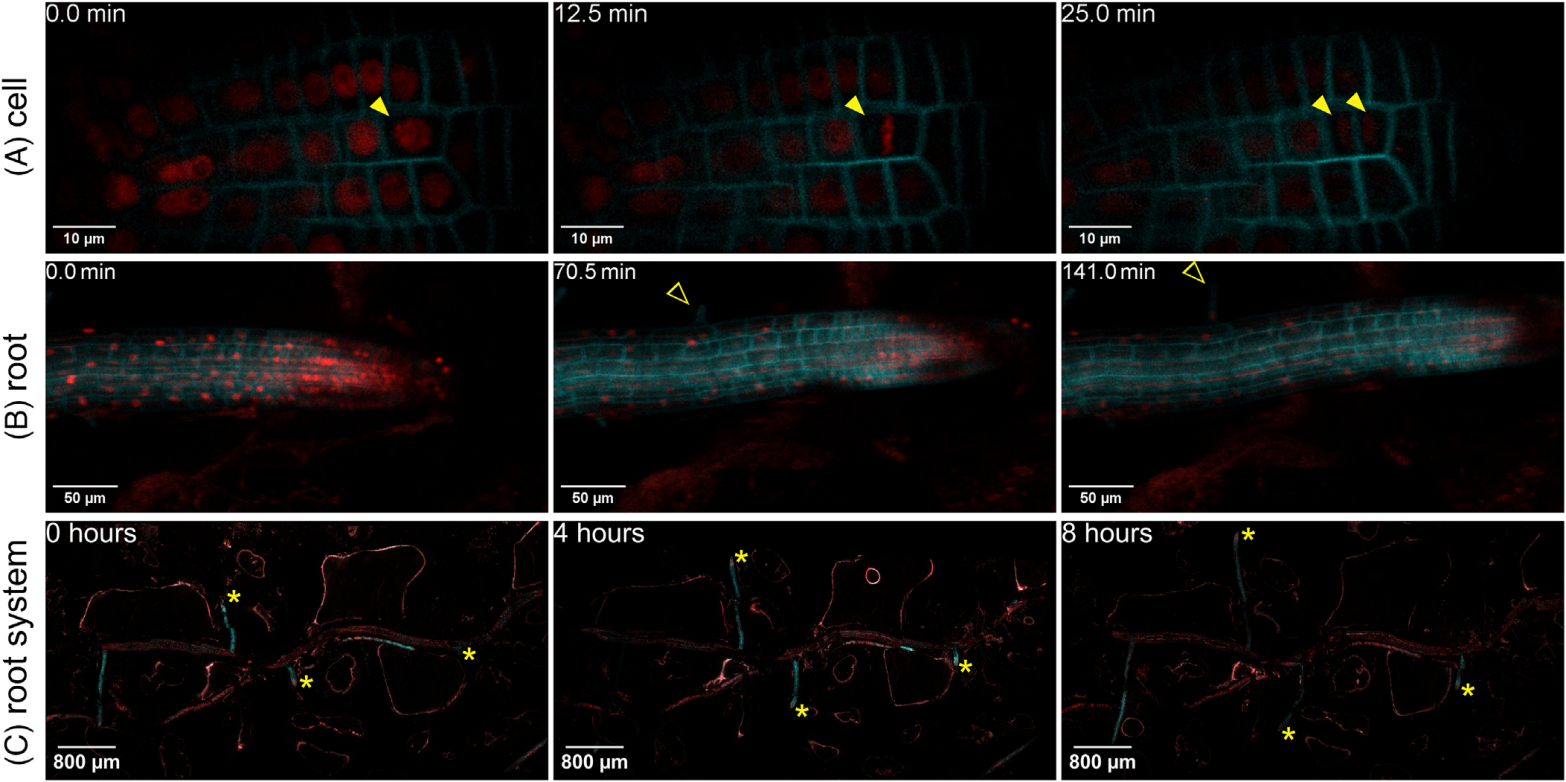
Nonmycorrhiza applications of AMSlides across spatiotemporal scales. *eCFP‐Lti6a; H2B‐mCherry* rice plants were grown in AMSlide 3 for 12 days before confocal imaging of (A) mitosis and cytokinesis of root tip cell (solid yellow arrowhead), (B) growth of root tip around soil particles and development of root hair (open yellow arrowhead), and (C) growth of fine lateral roots (yellow asterisks) from a large lateral root. (A) 30‐second scan intervals for 27 min, images are summed z projection of 2 µm volume. (B) 45‐second scan interval for 150 min, images are summed z projections of 24 µm volume. (C) Images captured at 4‐h intervals for 8 h, images are stitched tile‐scans of single optical planes. Cyan = eCFP, red = mCherry.

The temporal context of underground processes is not just intrinsic to AM studies, but also root research more broadly. To investigate the AMSlides' suitability for non‐AM applications, the plasma membrane and chromatin coexpression reporter *eCFP‐Lti6a; H2B‐mCherry* ^50^ was grown in AMSlide 3 for 12 days before imaging at various spatiotemporal scales. Nuclear dynamics and cell division over seconds to minutes, root tip growth over minutes to hours, and root network expansion over multiple hours were all successfully captured (Figure 6, Movies S6 and S7).

## 4 DISCUSSION

### 4.1 An affordable and adaptable live‐imaging system

Due to the similar shape and dimensions of the AMSlides to a standard microscope slide, they are compatible with commonly used microscope apparatus. Their versatility in coverslip position, growth substrate, and chamber orientation makes them adaptable to many different modes of microscopy, such as upright or inverted systems, transmission or epifluorescence, confocal or widefield (Figure 1, File S6), unlike the only previous noninvasive systems which were restricted to inverted, epifluorescence‐based hardware.^18^, ^49^ While not tested, AMSlides would also be compatible with multiphoton microscopy for deeper‐root imaging, or spinning disk microscopy for capturing faster dynamics.

The 3D‐printable design, easy‐to‐obtain materials, and no requirement for sterile conditions make the AMSlide a simple and affordable system for live‐imaging AM symbiosis. However, the time lapses of nonsymbiotic roots in this work, from the scale of chromatin dynamics to root system development, highlights the applicability of AMSlides beyond AM symbiosis research (Figure 6). The inclusion of natural growth substrates, such as sand or soil, may offer a more representative system for studying root development, plant–pathogen interactions^59^ and rhizosphere dynamics^60^ than currently available devices.^61^, ^62^, ^63^, ^64^

### 4.2 AM symbiosis can be live‐imaged, noninvasively at high resolution

The AMSlides enable truly noninvasive live imaging of AM colonisation. The in situ imaging allows more reliable observations than high‐disturbance methods involving uprooting the plants, excising or sectioning. Despite being small, the AMSlides facilitated comparable plant growth and AM colonisation to conventional pot systems and no effects of photo‐toxicity were observed (Figures S1 and S2). They likely represent a more ‘natural’ scenario than previously used growth pouches or agar, offering the heterogeneous structure and moisture properties of soil/sand. This is particularly relevant for rice, which on agar rarely develops the large lateral roots that preferentially host AM fungi.^65^ Additionally, using an entire, intact plant is likely more physiologically relevant than root organ cultures, which lack a shoot, or hairy roots, considering the known roles of shoot‐borne signals in AM regulation.^66^

The AMSlides achieve better imaging resolution than previously reported noninvasive imaging set‐ups. Subcellular details such as arbuscule domains can be resolved (Figure 2B), which are intrinsic to understanding arbuscule development, nutrient exchange and colonisation dynamics. The improved resolution is partly because many previous systems were only compatible with widefield fluorescence microscopy, comparatively lower in resolution than confocal laser scanning microscopy.^18^

The image resolution achieved with AMSlides is even comparable to standard root excision and slide‐mounting methods (Figure 2B and C). While the roots of pot‐grown plants grow in three dimensions and therefore rarely sit flat on a microscope slide, the roots of AMSlide plants grow adjacent to the coverslip, reducing the focal distance and required working distance of the objectives.

### 4.3 AMSlide brings the (spatio‐)temporal context to AM cell biology studies

The AMSlides enable AM processes to be monitored over a wide range of timeframes. This is important for understanding AM symbiosis, where it can be similarly desirable to capture fast processes, such as trafficking of proteins to the symbiotic interface or changes in nutrient levels over minutes to hours, as it is to follow the much slower progression of colonisation over days to weeks.

The observations made using AMSlides are largely consistent with previous studies, supporting their reliability. For example, the mobility of the SCAMP‐puntae and transvacuolar strands around collapsed arbuscules mirrors time‐lapses made by Kobae and Fujiwara.^49^ The timing of arbuscule development and collapse recorded in AMSlides – commonly 1–2 days to grow, 1–2 days (but sometimes up to 4 days) in cell‐filling state, then rapid collapse followed by 3–5 days to fully disappear – concurs with time‐lapse measurements made by Kobae et al.,^18^, ^49^ as well as estimations made by Alexander et al.^67^, ^68^ The measurement of colonisation progression, ∼400 µm/day in the example here, also concurs with earlier observations.^18^ But unlike previous studies, subcellular detail is visible at every timepoint. Consequently, precise developmental stages and fine‐scale processes could be monitored throughout AM colonisation, revealing for example the different temporal dynamics of coils compared to arbuscules of *G. margarita* (Movie 3, Figure S5b), or different *eGFP‐SCAMP* expression dynamics throughout arbuscule development (Figure 4).

### 4.4 Future applications of the AMSlide

The AMSlide was developed and optimised using *eGFP‐SCAMP*‐expressing rice as a useful highlighter of symbiotic structures. However, the future applications are multitude. Combining the AMSlide system with genetically encoded reporters for genes relating to symbiotic signalling, intracellular colonisation, nutrient exchange or the ever‐expanding suite of biosensors should allow the temporal context of these processes to be uncovered. Vital stains further broaden the cellular processes that could be monitored.

The selection of plants and fungi successfully imaged in the AMSlides include monocot and dicot plant examples, and AM fungi from the *Glomerales* and *Gigasporales*, which show distinct growth and development (Figure S5). It is therefore likely that AMSlides are compatible with many further plant–AM fungal partnerships, potentially shedding light on different symbiotic strategies, functions and nutritional outcomes.

The ability to image root colonisation and external hyphae over time in AMSlides should allow investigation of the relationship between internal and external hyphal activities (Figure 5). For example, how do pre‐ and postsymbiotic fungal behaviours, recently revealed via the *Obstacle chip* and *AMF‐SporeChip*,^16^, ^48^ relate to within‐root colonisation dynamics? Monitoring AM symbiosis over time could unearth previously hidden differences in AM colonisation dynamics, such as arbuscule development, lifetime and rate of colonisation. How do these parameters change with different biotic situations (e.g. different plant or fungal species and genotypes) or abiotic conditions (e.g. stresses, nutrient levels, light availability)?

Using the AMSlides, we can better uncover the temporal ‘black box’ of AM symbiosis, which has been taking place in the roots of most plant species across the globe for at least the past 450 million years.^3^ It is hoped the ability to observe the interaction between plants and AM fungi in a more natural state, nondisruptively, over time, will improve our fundamental understanding of the intrinsically dynamic AM symbiosis.

## AUTHOR CONTRIBUTIONS

JM designed and optimised the AMSlide, performed most experiments and wrote the manuscript. BS performed FRAP experiments. UP edited the manuscript.

## Supporting information

Supporting Information

Supporting Information

Supporting Information

Supporting Information

Supporting Information

Supporting Information

Supporting Information

Supporting Information

Supporting Information

Supporting Information

Supporting Information

Supporting Information

Supporting Information

Supporting Information
