# Supplementary material for: The AMSlide for noninvasive time‐lapse imaging of arbuscular mycorrhizal symbiosis
Source: J Microsc. 2024 May 15;297(3):289–303. doi: 10.1111/jmi.13313 (PMC11808451; doi:10.1111/jmi.13313)
Supplement: Supplementary file 1 — Supporting Information [file JMI-297-289-s002.docx]

**SUPPORTING FIGURES, MOVIES and FILES**

**Supporting Figures S1 – S5**


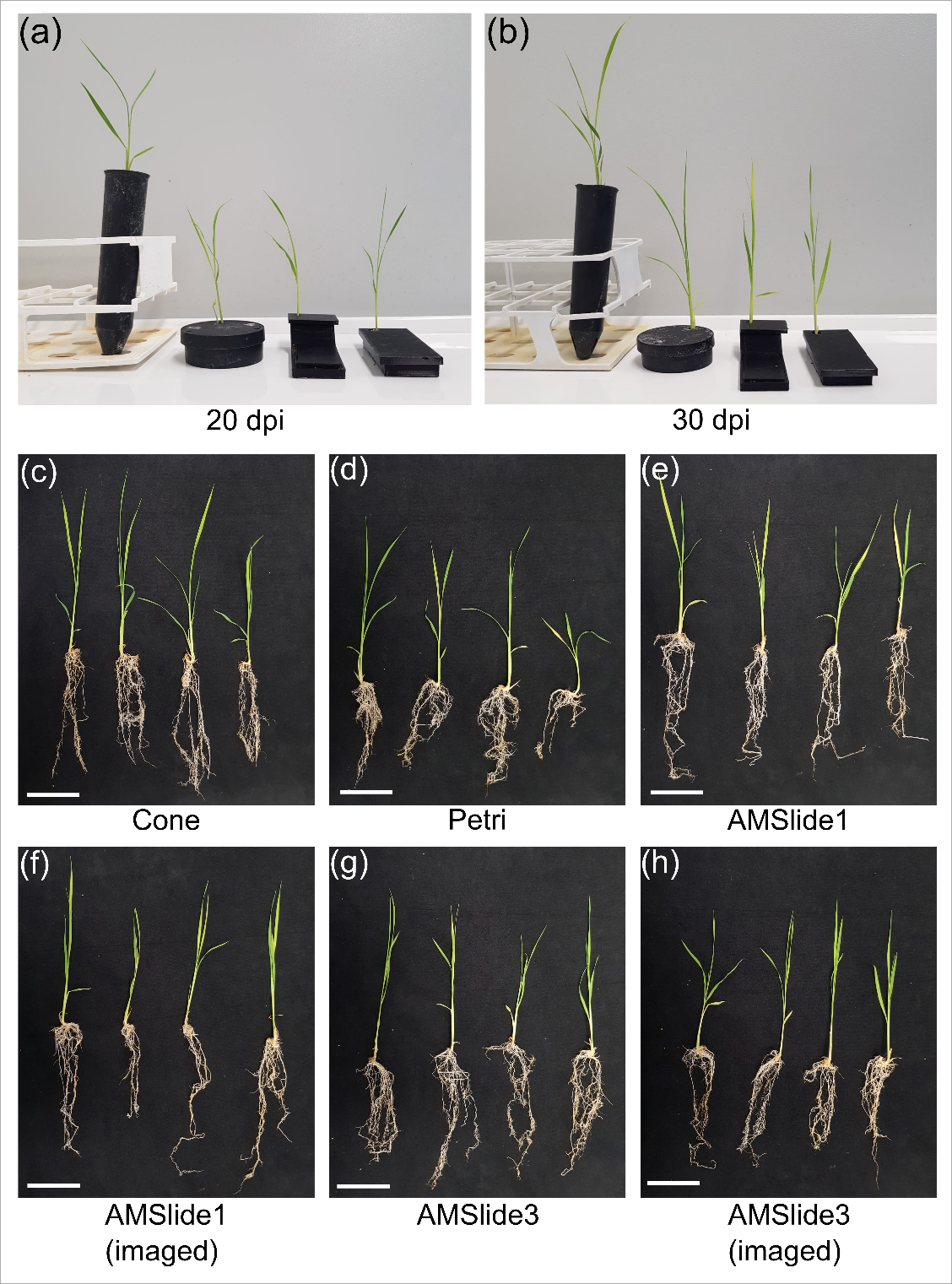


**Figure S1.** Rice grown in AMSlide chambers compared to pot and cone systems. (a-b) Representative images of *eGFP-SCAMP* rice colonised by *R. irregularis* growing in cone, petri dish, AMSlide 1 and AMSlide 3 systems at (a) 20 days post inoculation (dpi) and (b) 30 dpi. (c-h) Root and shoot systems of plants at the 30 dpi harvest from (c) cone, (d) petri dish, (e-f) AMSlide 1, and (g-h) AMSlide 3. (f,h) AMSlide-grown plants that were imaged by confocal laser scanning microscopy daily between the harvest timepoints. Scale bars = 50 mm.

**
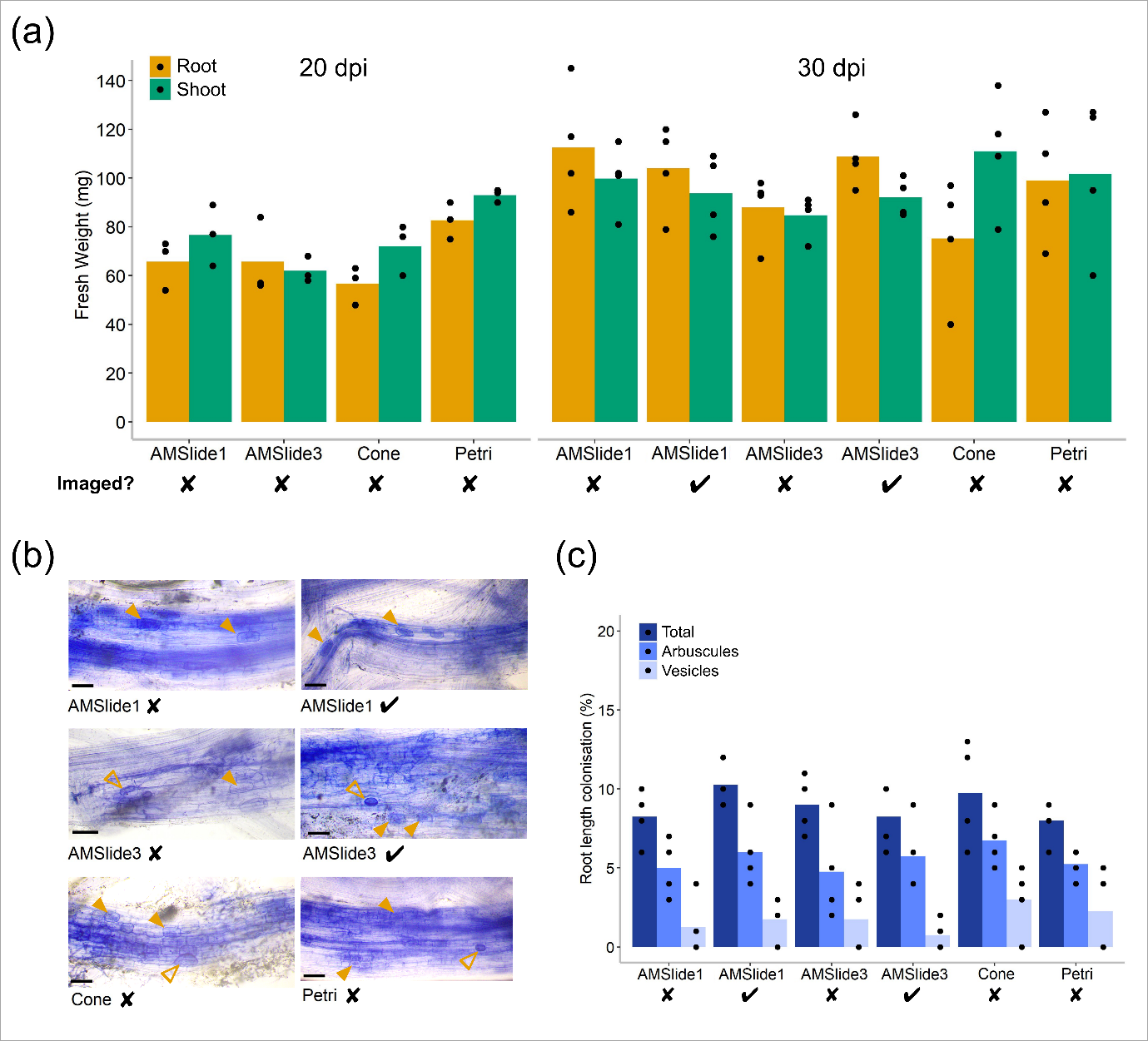
**

**FIGURE S2.** Comparison of growth and AM colonisation between AMSlide chambers and conventional growth set-ups. (a) Shoot and root fresh weights of *eGFP-SCAMP* rice inoculated with *R. irregularis* grown in different set-ups for 20 and 30 days post inoculation (dpi). ✘ = plants that were not live-imaged, **✓** = plants that were imaged daily between the harvest timepoints. No statistical difference in shoot or root fresh weights was found between chamber types or imaged/not imaged plants (Kruskal Wallis rank sum test, *p > 0.05*). (b) Representative micrographs of colonised roots from rice grown in different set-ups at 30 dpi. Orange solid arrowheads = arbuscules, open arrowheads = vesicles. Scale bars = 100 µm. (c) Percent of root length hosting any internal colonisation (‘Total’), arbuscules or vesicles at 30 dpi of rice grown in different set-ups. No statistical difference in presence of any colonisation structure was found between chamber types or imaged/not imaged plants (Kruskal Wallis rank sum test, *p > 0.05*).


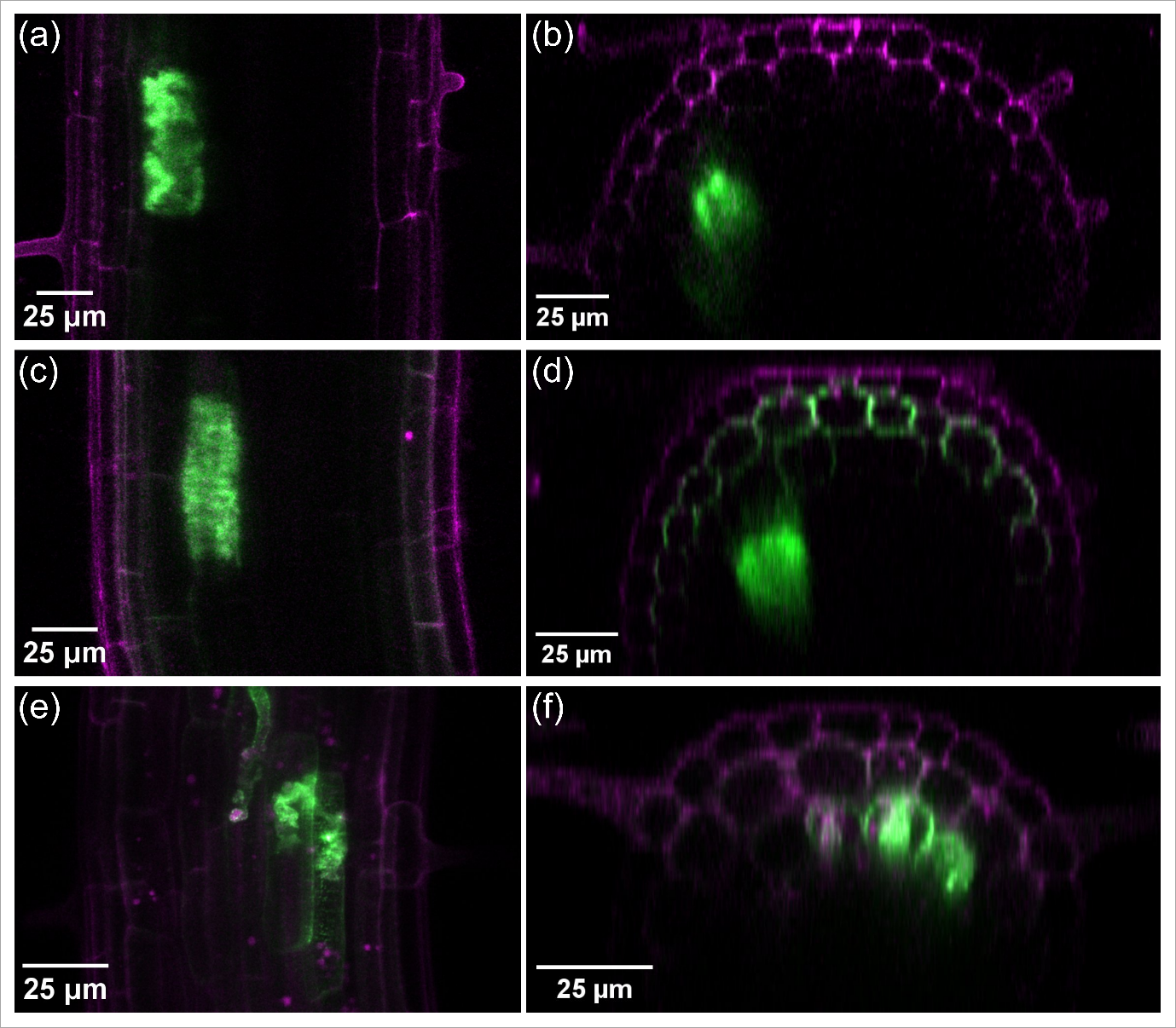


**Figure S3.** Transverse and radial views of *eGFP-SCAMP* rice colonised by *R. irregularis* showing achievable imaging depth in AMSlides. (a,c,d) Selection of lateral roots imaged longitudinally and (b,d,f) corresponding optical resliced images showing depth of imaged-arbuscules within roots, namely 4 cell layers in (d), 3-4 cell layers in (b), and 3 cell layers in (f). All images are summed z projections of 20 µm volume. Green = eGFP, magenta = autofluorescence.


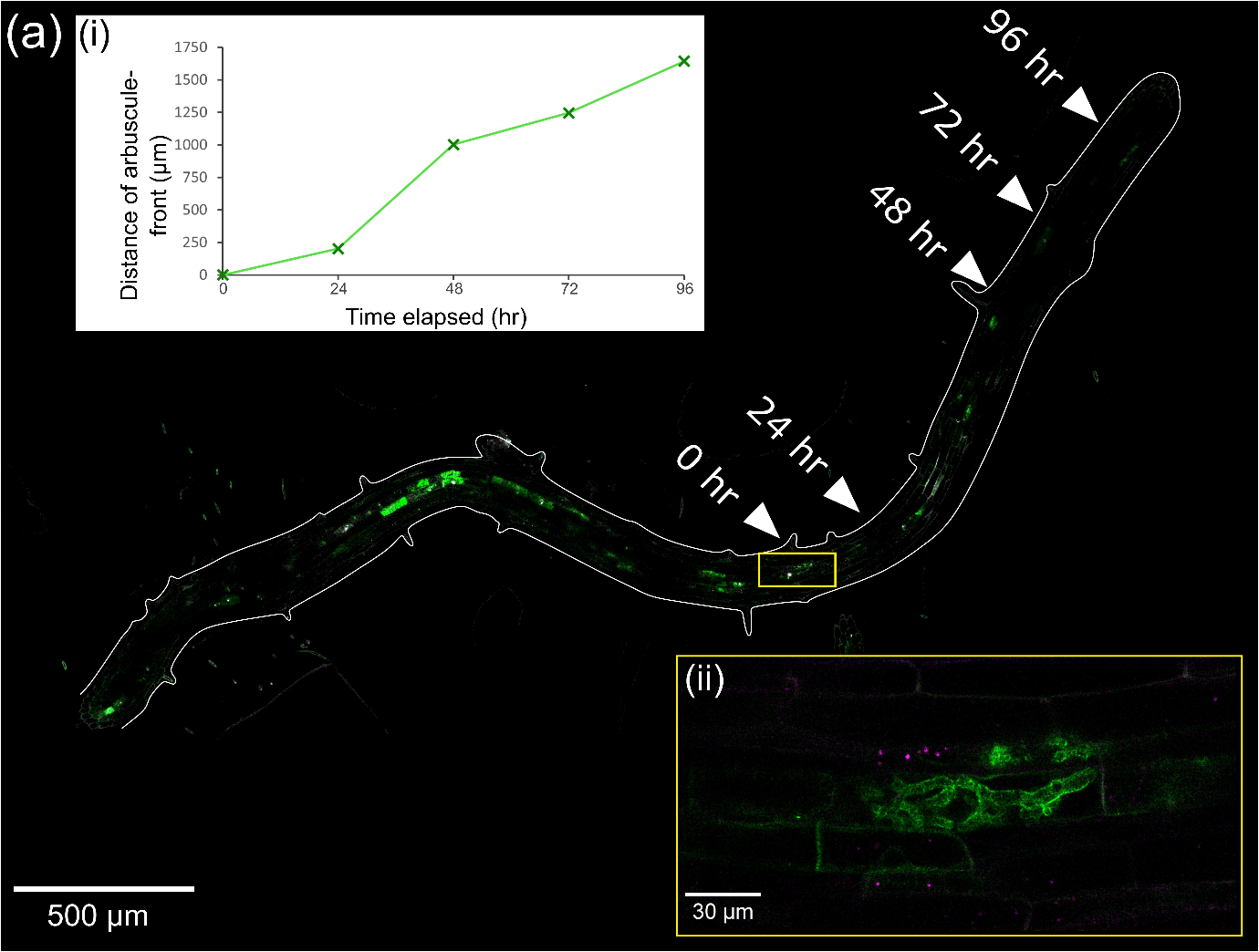


**Figure S4.** The rate of colonisation progression monitored and quantified in the AMSlide system. (a) A merged tile-scan image (single optical plane) of an *eGFP-SCAMP* rice root colonised by *R. irregularis* grown in AMSlide 3. White arrowheads mark the positions of the ‘arbuscule front’ (arbuscule at the growing edge of fungal colonisation) visible at each timepoint. (i) Inset graph plots the rate of colonisation progression along the root over time. (ii) Enlargement of yellow box in (a) shows image resolution achieved, depicting a young, coarsely-branched arbuscule (maximum intensity projection). Green = eGFP, magenta = autofluorescence.


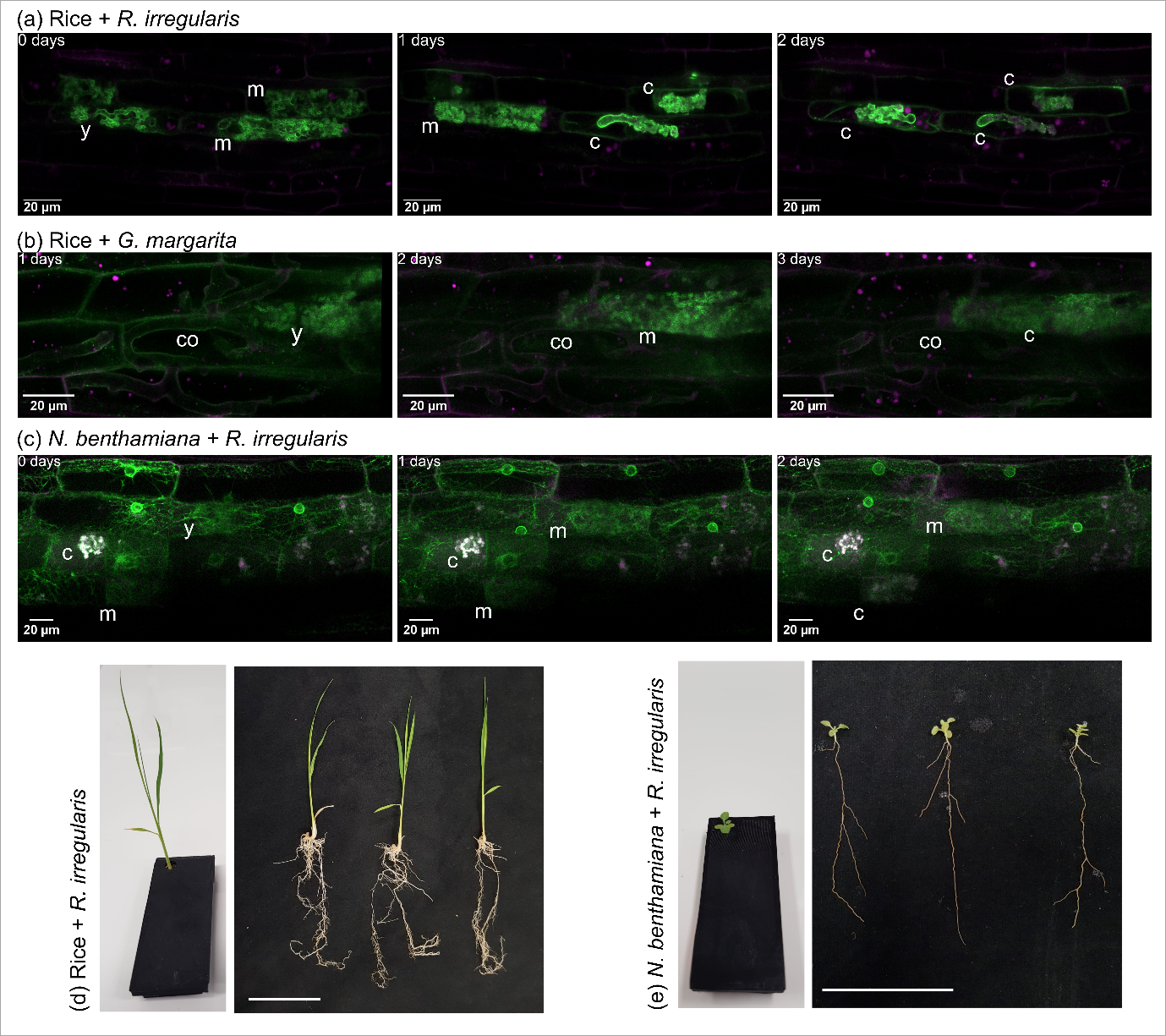


**Figure S5.** Timelapse imaging of different plant and fungal partners in AMSlides. (a) *eGFP-SCAMP* rice colonised by *R. irregularis* showing arbuscule development and collapse. (b) *eGFP-SCAMP* rice colonised by *G. margarita* showing stable coils alongside a developing/collapsing arbuscule. (c) *GFP16C N. benthamiana* colonised by *R. irregularis* showing arbuscule development and collapse. All plants imaged in AMSlide 3 at daily intervals after 20 days post inoculation. Maximum intensity projections. y = young arbuscule, m = mature arbuscule, c = collapsed arbuscule, co = coil, green = GFP, magenta = autofluorescence. (d-e) Example growth of (d) rice and (e) *N. benthamiana* in AMSlide 3 at 20 days post inoculation with *R. irregularis.* Scale bars = 50 mm.

**Supporting Movies S1 – S6**

**Movie S1.** Timelapse movie capturing fast dynamics of eGFP-SCAMP in *R. irregularis-*colonised rice root cells hosting young and collapsed arbuscules. Plants grown in AMSlide 3. Images were taken at 1-minute intervals for 45 minutes. Maximum intensity projection of entire arbuscule. Green = eGFP, magenta = autofluorescence.

**Movie S2.** Timelapse movie capturing collapse of arbuscules in *eGFP-SCAMP* rice root colonised by *R. irregularis.* Plants grown in AMSlide 3. Images were taken at 4-hour intervals for 12 hours. Maximum intensity projections. Green = eGFP.

**Movie S3.** Timelapse movie capturing growth of fungal storage vesicle in *eGFP-SCAMP* rice root colonised by *R. irregularis*. Plants grown in AMSlide 1. Images were taken at 24-hour intervals for 4 days. Micrographs are maximum intensity projections. Green = eGFP.

**Movie S4.** Timelapse movie of arbuscule growth and collapse in *eGFP-SCAMP* rice root colonised by *R. irregularis,* grown in AMSlide 3. Annotations shown in Figure S5. Micrographs are maximum intensity projections. Green = eGFP, magenta = autofluorescence.

**Movie S5.** Timelapse movie of arbuscule growth and collapse in *GFP16C N. benthamiana* colonised by *R. irregularis.* Plants grown in AMSlide 3. Annotations shown in Figure S5. Images are maximum intensity projections. Green = GFP, magenta = autofluorescence.

**Movie S6.** Timelapse movie of mitosis and cytokinesis of a root tip cell in *eCFP-Lti6a ; H2B-mCherry* rice grown in AMSlide 3. Annotations shown in Figure 6a. Images were taken at 30 second intervals for 27 minutes. Micrographs are summed Z projections of 2 µm volume. Cyan = eCFP, red = mCherry.

**Movie S7.** Timelapse movie of root growth in *eCFP-Lti6a ; H2B-mCherry* rice grown in AMSlide 3. Annotations shown in Figure 6b. Images were taken at 45 second intervals for 150 minutes. Micrographs are summed Z projections of 24 µm volume. Cyan = eCFP, red = mCherry.

**Supporting Files S1 – S6**

**File S1.** 3D print file for AMSlide 1 imaging chamber (.stl file)

**File S2.** 3D print file for AMSlide 2 imaging chamber lid 1 (.stl file)

**File S3.** 3D print file for AMSlide 2 imaging chamber lid 2 (.stl file)

**File S4.** 3D print file for AMSlide 3 imaging chamber (.stl file)

**File S5.** 3D print file for AMSlide 3 imaging chamber lid (.stl file)

**File S6.** Supplementary protocol for AMSlide assembly and use (PDF file)
