# Supplementary material for: The AMSlide for noninvasive time‐lapse imaging of arbuscular mycorrhizal symbiosis
Source: J Microsc. 2024 May 15;297(3):289–303. doi: 10.1111/jmi.13313 (PMC11808451; doi:10.1111/jmi.13313)
Supplement: Supplementary file 2 — Supporting Information [file JMI-297-289-s006.pdf]

File S6. Supplementary Protocol for the AMSlide

1. Choice of chamber

- There are four main chamber set-ups for the AMSlide, each with different applications (see Figure 1 in the main text).

- **AMSlide 1**: This chamber has a coverslip sealed to the lower side, removable lids on the upper side. Compatible with inverted epifluorescence microscopy of plant roots grown in an opaque substrate (e.g. soil or sand). Additionally can be used for transmitted light imaging if soil is washed from on top of the coverslip prior to imaging. Grown at 30° angle.

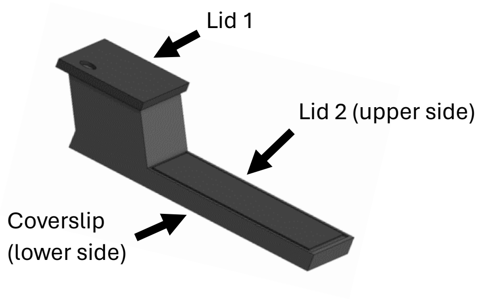

- **AMSlide 2:** Uses the same chamber set-up as AMSlide 1, but with a transparent growth substrate (only suitable for plant-fungal combinations that engage in AM symbiosis in water/agar). Compatible with inverted epifluorescence and transmitted light microscopy. Grown at 30° angle.

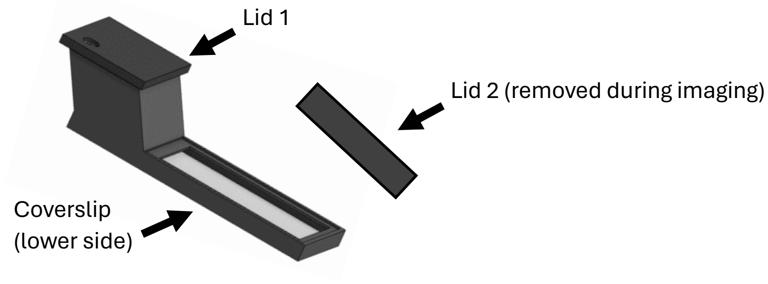

- **AMSlide 3:** A simpler chamber with coverslip sealed to lower side, removable lid on upper side. Compatible with inverted epifluorescence microscopy of plant roots grown in an opaque substrate (e.g. soil or sand). Similar use-case to AMSlide 1 without the ability to do transmitted light microscopy. Grown at 30° angle.

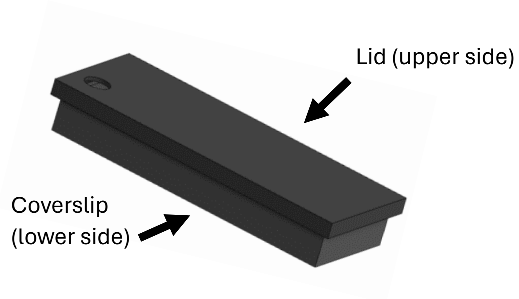

- **AMSlide 4:** Same chamber as AMSlide 1 but with the coverslip sealed to the upper side and Lid 2 sealed to lower side. Lid 1 taped on (removable for watering). Compatible with upright epifluorescence microscopy of plant roots grown in an opaque substrate (e.g. soil or sand). Grown in vertical orientation.

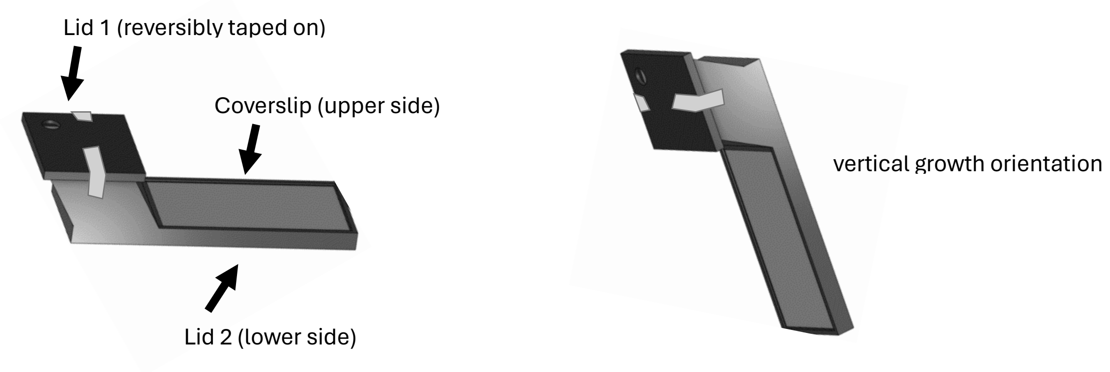

2. 3D printing

- A 3D printer with dual nozzles is required (e.g. an Ultimaker Cura S3)
- Printing filaments: black PETG used with an AA0.4 print core (for chamber and lids) and natural PVA with a BB0.4 print core (for dissolvable support)
- 3D printing parameters in Cura software: fine 0.1mm resolution, units = mm, support activated (extruder 2), adhesion activated

- Print chambers and lids
- Trim excess plastic off chamber and lids with scalpel and leave to soak overnight in water to dissolve the PVA supports
- Leave to air dry
- Clean surfaces with 70% ethanol

3. Coverslip attachment

- Squirt a line of silicone (transparent Dowsil 732) around the inset of the chamber where the coverslip will be attached: lower side for AMSlide 1, 2 and 3, upper side for AMSlide 4
- Insert the coverslip (24 x 50mm #1.5H coverslip)
- Scrape off any excess silicone to create a flat surface
- * For AMSlide 4: Repeat with the 3D printed Lid 2 on the lower side*
- Leave to cure for 24 hours
- Rinse with reverse osmosis (RO) water

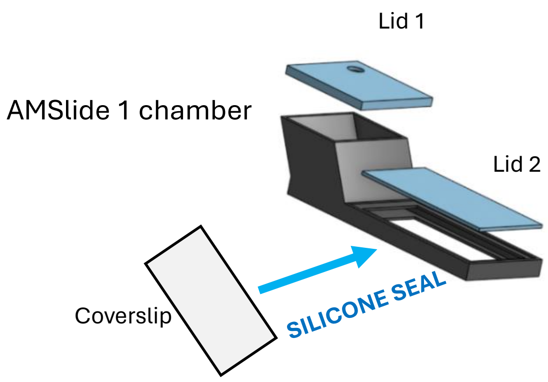

4. Choice of inoculation

- Different inoculation strategies can be used depending on the experiment
- **Spore inoculation**: spores are mixed into the AMSlide sand at 100 spores/chamber – allows early colonisation events to be monitored, but also the slowest set-up (~20 days to first colonisation with rice and *R. irregularis*)
- **Crude inoculum**: crude inoculum mixed into the AMSlide sand at 5% (v/v) – allows early colonisation events to be monitored, but faster than just spores (~12 days to first colonisation with rice and *R. irregularis*)
- **Nurse plant inoculation**: seedlings can be pre-colonised using a nurse plant system (see main text section 2.3) before transferring to AMSlide chamber. Gives fast, robust colonisation (from ~7 days post-planting with rice) but cannot observe early colonisation events
- ***Spore/ crude concentrations should be adjusted depending on species and inoculum age or viability***
- ***Inoculum positioning can be adjusted depending on research question. e.g. positioning inoculum only over coverslip to capture first colonisation events***

5. Planting

- Add a thin layer of silica sand (with or without fungus, depending on inoculation strategy) to the base of the chamber
- Transfer the seedling to the non-coverslip end of the AMSlide
- Fill chamber with silica sand
- Water with RO water until sand is just damp
- Add lid(s)
- *For AMSlide 4: Lid 1 will need to be taped on*

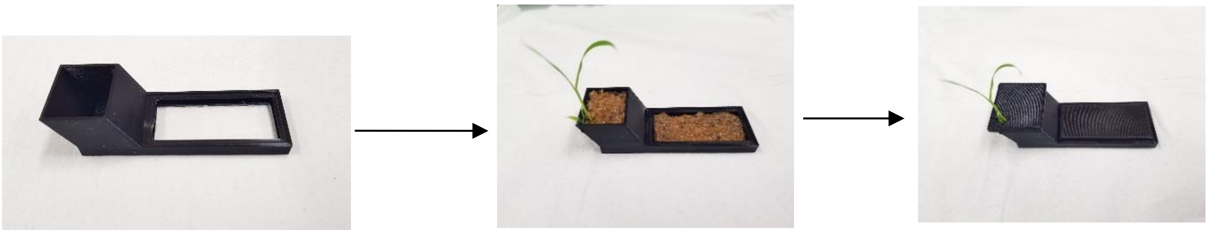

AMSlide 1 example

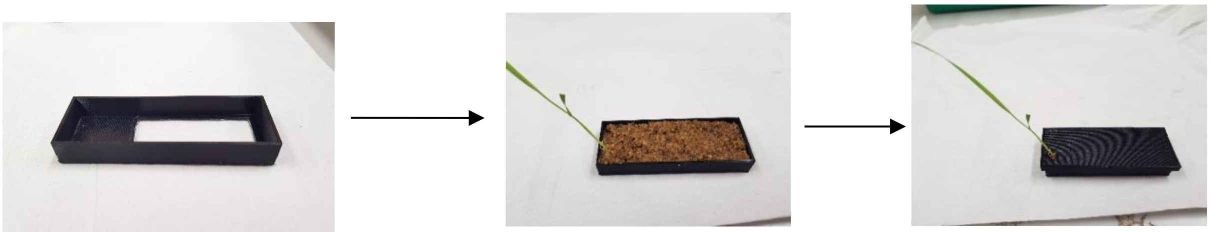

AMSlide 3 example

6. Watering and growth conditions (rice)

- Place chambers on tissue paper/lens tissue in a tray to prevent scratching the coverslip
- Tilt tray at 30° to aid downwards root growth
- *For AMSlide 4: prop chamber in vertical orientation to guide root growth against coverslip*
- Add prop lid to tray to prevent drying
- Place in growth chamber with following conditions:
    - Day: 12 hour, 300 uE light, 28°C, relative humidity 65%
    - Night: 12 hour, dark, 20°C, relative humidity 65%

- Water every other day, always watering until sand is *just* damp
- For the first week use RO water only
- From week two onwards, alternate watering with ½ strength Hoaglands (modified P_i = 25 μM) and RO water
- Monitor progress until roots visible through coverslip

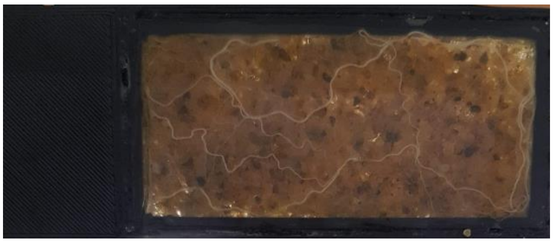

Example AMSlide 1 at 20 days post planting

7. Imaging

- Roots should appear in the imaging chamber window from 5-7 days after planting (for rice)
- Clean coverslip with 70% ethanol and lens paper
- Transfer to confocal microscope (inverted for AMSlide 1, 2, 3, upright for AMSlide 4)
- Screen roots for colonisation using a low magnification dry objective (10x or 20x), then use water immersion high magnification objective (e.g. 40x) for imaging
- Record stage XY coordinates and sketch root map (or tilescan overview of root e.g. Figure S4) to aid re-location of regions of interest
- Use as low laser power as possible e.g 4% was sufficient in this study
- After imaging, return AMSlide to growth chamber until next timepoint
- At next timepoint, apply identical imaging parameters before imaging (laser and detector settings, scan and format settings, zoom, rotation, line accumulations etc.)

8. Image analysis

- Images from different timepoints can be aligned in FIJI/ImageJ using the 'Align Image by line ROI' plugin
- Z-stack images require z-projecting (e.g. 'Max Intensity Z project' function) or a single channel should be chosen and 'Duplicate'd before aligning
- Multichannel images require splitting and aligning individually using the 'Split Channels' and 'Merge Channels' functions (a 'Macro' can be used to repeat alignment for each channel, saving the line ROI in the 'ROI Manager' and applying to each channel)
- The 'Concatenate' function can be used to combine aligned images into an *xyct* hyperstack
- Aligned images must be re-calibrated using a measurement taken from original .tiff image ('Measure' and 'Set Scale' functions)
- Overlayed scale bars ('Scale Bar') and timepoints ('Series Labeler') must be 'Flatten'ed before saving as animation
- Timelapse animations can be generated by 'Save As Animated Gif' or 'Save As .avi'
